# The Complete Plastid Genome of *Magnolia zenii* and Genetic Comparison to Magnoliaceae species

**DOI:** 10.3390/molecules24020261

**Published:** 2019-01-11

**Authors:** Yongfu Li, Steven Paul Sylvester, Meng Li, Cheng Zhang, Xuan Li, Yifan Duan, Xianrong Wang

**Affiliations:** 1 Co-Innovation Center for Sustainable Forestry in Southern China, Nanjing Forestry University, Nanjing 210037, China; liyongfu1994@outlook.com (Y.L.); limeng@njfu.edu.cn (M.L.); 18362928857@163.com (C.Z.); xuanli18851128817@163.com (X.L.); 2 College of Biology and the Environment, Nanjing Forestry University, Nanjing 210037, China; steven_sylvester@hotmail.com

**Keywords:** traditional Chinese medicine, chloroplast genome, microsatellite, comparative analysis, phylogeny

## Abstract

*Magnolia zenii* is a critically endangered species known from only 18 trees that survive on Baohua Mountain in Jiangsu province, China. Little information is available regarding its molecular biology, with no genomic study performed on *M. zenii* until now. We determined the complete plastid genome of *M. zenii* and identified microsatellites. Whole sequence alignment and phylogenetic analysis using BI and ML methods were also conducted. The plastome of *M. zenii* was 160,048 bp long with 39.2% GC content and included a pair of inverted repeats (IRs) of 26,596 bp that separated a large single-copy (LSC) region of 88,098 bp and a small single-copy (SSC) region of 18,757 bp. One hundred thirty genes were identified, of which 79 were protein-coding genes, 37 were transfer RNAs, and eight were ribosomal RNAs. Thirty seven simple sequence repeats (SSRs) were also identified. Comparative analyses of genome structure and sequence data of closely-related species revealed five mutation hotspots, useful for future phylogenetic research. *Magnolia zenii* was placed as sister to *M. biondii* with strong support in all analyses. Overall, this study providing *M. zenii* genomic resources will be beneficial for the evolutionary study and phylogenetic reconstruction of Magnoliaceae.

## 1. Introduction

*Magnolia zenii* Cheng is listed as a critically endangered species in the IUCN Red List of Threatened Species (www.iucnredlist.org) due to there being only one population of 18 individuals existing on the northern slopes of the Baohua Mountain in Jiangsu, China. The small population of *M. zenii* is found within a provincial reserve, but no specific protective measures are given to this species. The survival of the species remains highly uncertain, with no natural regeneration being observed and no information available regarding seed viability. Current research on the conservation of the species has focused on intra- and inter-specific competition [1,2], vegetative propagation [3], and endogenous hormone production [4,5]. Projections of climate change [6], with temperatures likely to increase while precipitation is predicted to decrease, mean that the species may face a greater risk of extinction.

Aside from its conservation value, *M. zenii* has been found to have potential pharmaceutical value, with tomentosin (sesquiterpene lactone), one of the volatile components isolated from seeds of *M. zenii*, possibly having applications in the treatment of inflammatory diseases [4,7,8]. The species also has great potential as an ornamental tree due to being early flowering.

Magnoliaceae, a family with approximately 335 species in 18 genera [9], is a core component of the Magnoliales and is distributed in temperate and tropical regions of eastern Asia and eastern North America to the Neotropics [10]. The Magnoliaceae family presents a classic Asian-North American disjunct distribution, with adequate fossil records all around Laurasia [10,11]. Members of the family also have relatively primitive flowers [12], which makes these taxa highly valuable for research in plant evolution and reproductive ecology [13,14,15]. There are widely differing opinions on the taxonomy of the Magnoliaceae, mainly regarding generic circumscription [16].

The genus *Magnolia* consists of about 70 plants distributed in temperate and tropical regions of eastern Asia and eastern North America to the Neotropics, among which 23 species are endemic to China [17]. This genus has been traditionally divided into subgenera *Magnolia* and *Yulania* Spach [18]. A further subgenus *Michelia* L. was proposed after preliminary phylogenetic studies revealed genus *Michelia* and subgenus *Yulania* were more closely related to each other than either one of them was to subgenus *Magnolia* [19]. Now, at least 11 well-supported sub-generic clades are recognized [20,21,22]. However, molecular research has found the genus to be paraphyletic, and support along the backbone of the *Magnolia* phylogeny is poor. This, coupled with the wide distribution of taxa within these subgeneric clades, alongside overlapping morphologies and no discrete boundaries in many morphological character states, has meant opinions on the subgeneric delimitation of *Magnolia* remain contentious.

The plastid genome (or “plastome”) of all angiosperms has a quadripartite structure consisting of a large single-copy (LSC) region, a small single-copy (SSC) region, and two copies of larger inverted repeats (IRs) [23]. While often referred to as the “chloroplast genome”, chloroplasts are just one type of plastid, along with leucoplasts, chromoplasts, and amyloplasts, which all develop from proplastids of an embryo and have an identical genome. The plastome varies in size from 75–250 kb and is highly conserved in terms of gene order and genome organization in flowering plants [24]. Hence, plastome sequences are widely used for exploring phylogenetic relationships in different plant groups [25,26]. However, some structural rearrangements have been discovered, such as gene or intron loss, and large inverted repeat (IR) expression [27,28]. Comparative analysis of plastomes can, thus, provide greater insight into the evolutionary history and interspecies relationships in the genus *Magnolia*.

In this study, we present the whole plastome sequence of *M. zenii* using Illumina sequencing technology and explore its relationships with other species within the genus. These results will be helpful for phylogenetic studies, while expanding horizons regarding the structural diversity of the plastome, as well as phylogenetic studies of Magnoliaceae species.

## 2. Results and Discussion

### 2.1. Genome Features and Guanine-Cytosine Content

**Figure 1:**
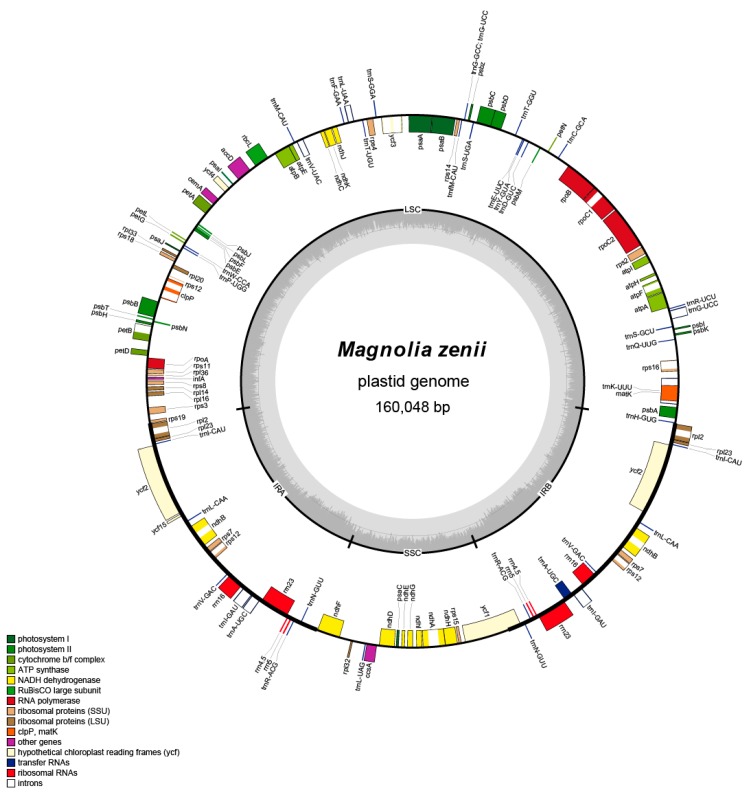
Plastid genome map of *M. zenii*. Genes inside the circle are transcribed clockwise, and those outside are transcribed counterclockwise. Genes of different functions are color-coded. The darker gray in the inner circle shows the GC content, while the lighter gray shows the AT content.

The full-length plastome of *M. zenii* was 160,048 bp, which is slightly larger than that of the closely-related species *Magnolia biondii* Pamp. (160,002 bp) (Table S1). The plastome length in other species of *Magnolia* ranges narrowly from 158,177 bp in *Magnolia liliiflora* Desr. to 160,183 bp in *Magnolia officinalis* Rehder & E.H. Wilson (Table S1) [29]. The plastid structure of *M. zenii* was a typical quadripartite circular molecule (Figure 1) that resembles the plastomes of the majority of angiosperms, which includes a large single-copy (LSC) region of 88,098 bp and a short single-copy (SSC) region of 18,757 bp divided into a pair of inverted repeat (IR) regions, IRA (26,596 bp) and IRB (26,596 bp) (Figure 1 and Table S1).

The percentage GC content of the total plastid DNA sequence is 39.2% (Table S1 and Table S2). The GC content of the LSC and SSC regions is 37.9% and 34.1%, respectively, while the IRA and IRB regions showed higher GC contents, with both at 43.2%, which is similar to other species of *Magnolia* such as *M. grandiflora* L. (Table S1) [30]. The higher GC content in the IR region compared with the SSC and the LSC regions is probably due to the presence of four ribosomal RNA genes duplicated in this region [31]. In fact, the overall GC content is often associated with the degree of primitiveness of a taxon: early-diverging lineages, such as magnoliids, tend to have a higher GC content compared to the average 35% GC content of most angiosperms [32]. The *M. zenii* plastome is AT-rich, which is consistent with other species of *Magnolia*; for instance, *M. grandiflora* (60.7%), *M. officinalis* (60.78%), *M. kwangsiensis* Figlar & Noot. (60.74%) [30], and other species like *Liriodendron tulipifera* L. (60.84%) [30], *Acer miaotaiense* P.C. Tsoong (62.12%), *Citrus sinensis* (L.) Osbeck (61.52%) and *Phellodendron amurense* Rupr. (61.60%) [31].

A total of 130 distinct genes were annotated, including 37 transfer RNAs (tRNA), 8 ribosomal RNAs (rRNA), and 84 protein-coding genes (Table 1 and Table S1). Five genes were duplicated in the IRs including *trnA-UGC*, *trnI-GAU*, *rps12*, *rpl2*, and *ndhB*. These genes are identical to those found in *M. grandiflora* [30]. The *rps12* is a specific trans-splicing gene of which the 5′-end exon is located in the LSC region, and the intron and 3′-end exon of the gene are situated in the IRs regions. Intron splicing is known to cause enhancement of gene expression [33], and spliceosomal introns are a landmark feature of eukaryotic nuclear genes [34]. Removal of these introns by the spliceosome can influence many other stages of mRNA metabolism, such as editing and polyadenylation of the pre-mRNA [35]. There were 17 intron-containing genes in the plastome of *M. zenii*, of which three genes (*rps12*, *ycf3*, and *clpP*) had two introns and the rest had one intron (Table S3). For many magnoliid species, such as *M. grandiflora*, *M. kwangsiensis*, *Drimys* J.R. Forst. & G. Forst. and *Piper* L., these same three genes (*rps12*, *ycf3*, and *clpP*) have two introns. Gene *ycf1* was the only pseudogene located in the junction between the SSC and IRB regions and was formed by the incomplete duplication of the normal copy *ycf1*. In general, the genes of *M. zenii* and related species are very similar, most likely due to the very slow rates of genome evolution in Magnoliaceae [29,36].

### 2.2. Codon Usage Bias

Based on theCoding sequence (CDS), the codon usage frequency (relative synonymous codon usage (RSCU) was estimated for the plastome of *M. zenii* (Table S4). All the protein-coding genes presented a total of 26,266 codons, leucine (2681 codons, approximately 10.20% of the total) being the most abundant amino acid in the *M. zenii* plastome. This is followed by isoleucine with 8.44%, while cysteine was rare with only 1.17%. Our findings match the trend reported across other angiosperm plastomes, which show leucine and isoleucine to be the most common [37,38]. Moreover, *Met* (AUG), *TER* (UGA), and *Trp* (UGG) are encoded by only one codon, which means they showed no codon bias. Besides that, almost all of the A/U-ending codons had RSCU values of more than one (RSCU > 1), whereas the C/G-ending codons had RSCU values of less than one.

### 2.3. Repeat Sequences

Microsatellites are chosen as ideal genetic markers in plant molecular studies such as *Papaver rhoeas* L. [39], *Lilium henrici* Franchet [37], and *Rosa chinensis* var. *spontanea* (Rehder & E.H. Wilson) T.T. Yu & T.C. Ku [38], because of their high variability. Microsatellites are usually ≤15 bp-long tandem repeat DNA sequences and are distributed throughout the genome [40]. The generally uniparental mode of plastid inheritance, which is usually maternal inheritance in angiosperms and paternal inheritance in gymnosperms, makes them powerful tools to elucidate relative contributions of seed and (or) pollen flow [41] to the genetic structure of natural populations of seed plants such as *Quercus* L. [42]. In this study, simple sequence repeats (SSRs) were detected in the plastome of *M. zenii* (Table S5). A total of 37 SSRs were identified. No pentanucleotide repeats were found. Mononucleotide SSRs were the richest (72.29%), and the mononucleotide A and T repeat units occupied the highest portion (96.67%). Our findings agree with the observation that plastid SSRs are generally composed of polyadenine (poly A) and polythymine (poly T) and rarely contain tandem guanine (G) and cytosine (C) repeats [43]. In addition, we found that mononucleotide, dinucleotide, and trinucleotide repeats were composed of a higher level of A or T; this contributed to a bias in base composition, which was consistent with the overall A-T richness (60.7%) of the plastome. The bias may have been caused by the ability of A to convert to T more readily than G-C in the genome [44].

A total of 50 long repeats were discovered in the *M. zenii* plastome (Table S6). This included 35 forward (F) repeats and 15 palindromic (P) repeats, while no tandem repeats were found. Among these, 35 forward repeats were 16–57 bp in motif length, and 15 palindromic repeats were 17–21 bp long. Most of the long repeats were located in the *ycf* gene and intergenic spacers (IGS). As with the SSRs, *M. zenii* also contained a lower number of long-repeat elements compared with other species. The presence of these repeats indicates that the locus is likely to be a crucial mutation hotspot in the genome because the repeat sequences allow sequence variations and genome rearrangements through slipped-strand mispairing and improper recombination [45].

### 2.4. Comparative Analysis of Plastomes of Magnolia Species

**Figure 2:**
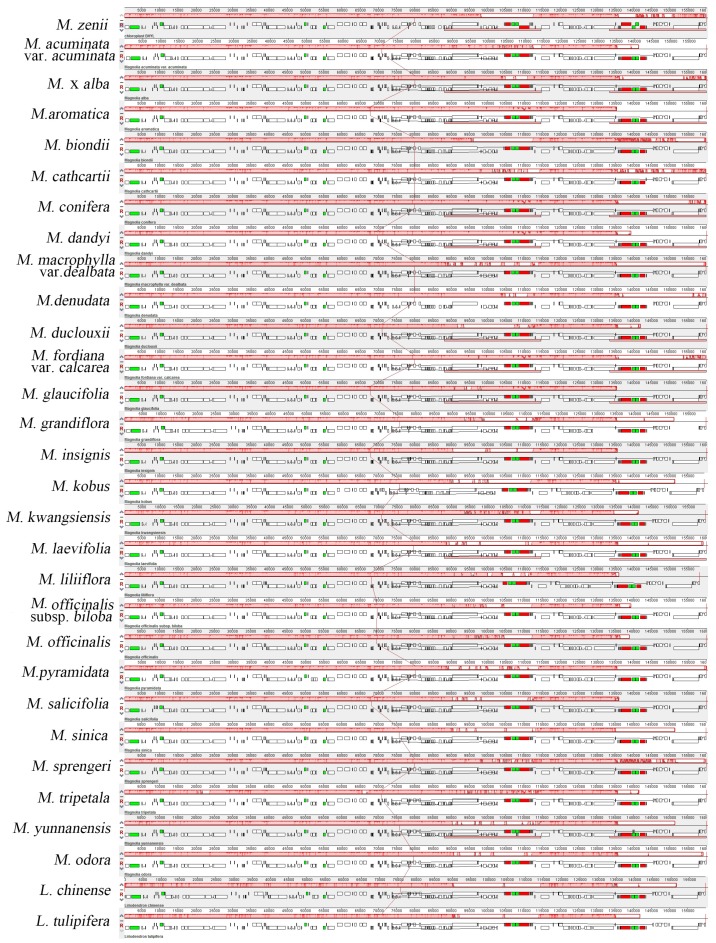
Mauve (Multiple Alignment of Conserved Genomic Sequence With Rearrangements) [47] alignment of plastid genomes of 28 species of *Magnolia* and two species of *Liriodendron*. The *M. zenii* genome is shown at the top as the reference genome. Within each of the alignments, local collinear blocks are represented by blocks of the same color connected by lines.

Complete genome alignment using Mauve software was done for 28 species of *Magnolia* (Table S1) and two species of *Liriodendron* (Figure 2). Whole-genome alignment of the plastomes revealed a common syntenic break at inverted repeat regions (∼90,000–115,000; ∼135,000–145,000), which also showed the highest variation in gene structure among the aligned plastomes. In general, the locally-collinear blocks (LCBs) depicted all these species to maintain a consistent position and direction in most of the genes, and no rearrangement or inversion events were found. Some other studies have suggested homology in plastome organization and no gene rearrangements in many plant groups [32,46], and our findings support their conclusions.

Comparison of DNA sequences from 30 species of Magnoliales is a fundamental method for identifying functional elements in genomes. The LAGAN (Limited Area Global Alignment of Nucleotides) [48] global alignment indicated that the IR regions are more conserved than the LSC and SSC regions (Figure S1). Rates of nucleotide substitution were previously shown to be several times slower in the plastid IR regions compared with SC regions in other angiosperms, suggesting that IR regions provide enhanced copy-correction activity [49]. Gene conversion has been shown to be biased towards copy-dependent repair mechanisms [50]. This phenomenon is possibly due to error correction occurring via gene conversion between Irs [51]. Furthermore, within the LSC and SSC regions, coding regions were more conserved than non-coding regions among the 30 plastomes. However, significant variations were found in coding regions of some genes including *matK*, *trnH-psbA*, *ndHh*, *ycf1*, and *ycf2*. In Orchidaceae, *ycf1* is identified as more variable and informative than *matK* at the species level, while also being easier to align than *ITS* genes [52]. *ycf2* is the largest plastid gene in angiosperms, although some species such as rice and maize [53] are reported to have independently lost this gene. The function of *ycf2* is largely unknown, but it appears to provide a good phylogenetic signal for resolving problems in plant phylogenies due to its long sequence length and low rate of nucleotide substitution [54]. Therefore, *ycf1* and *ycf2* may be potentially useful for phylogenetic research in *Magnolia*.

### 2.5. Junction Characteristics

**Figure 3:**
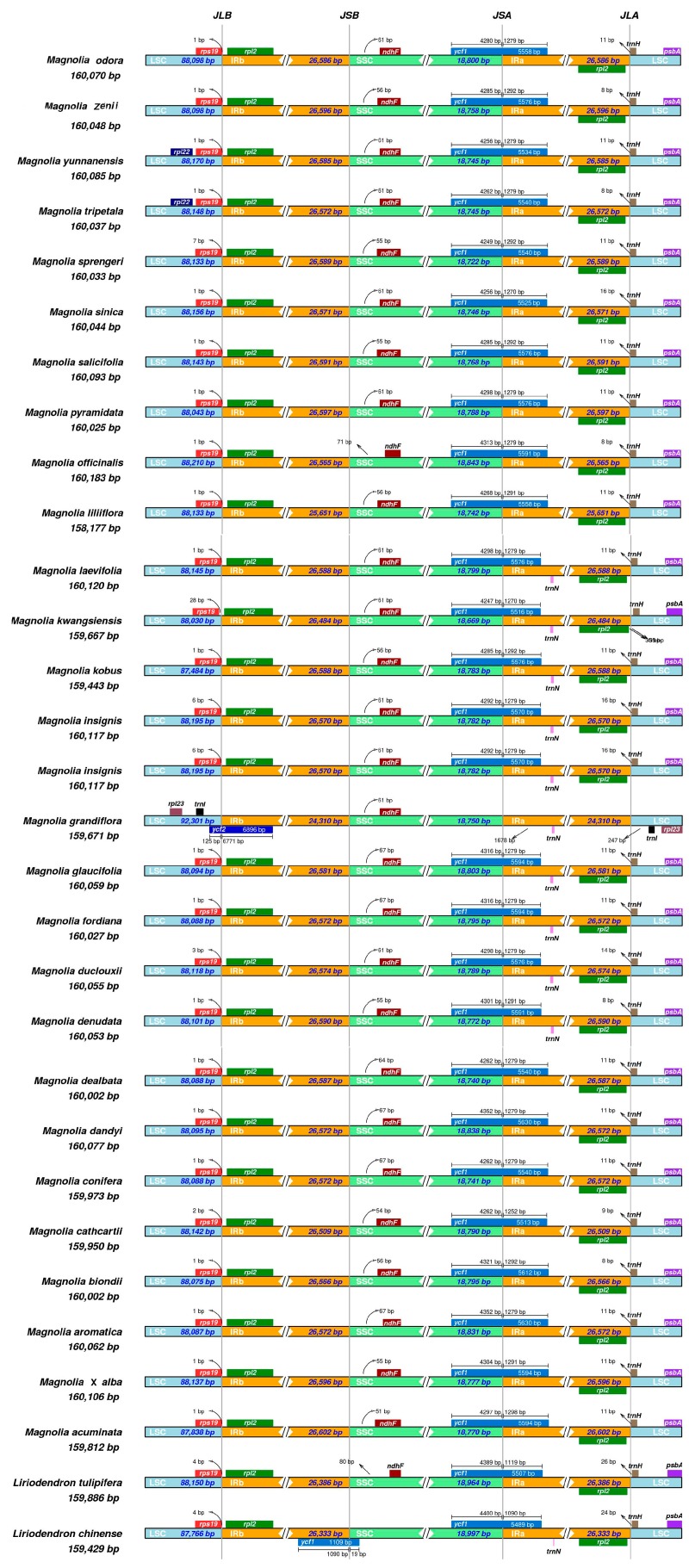
Comparison of the large single-copy (LSC), short single-copy (SSC), and inverted repeat (IR) regions in plastomes of 27 *Magnolia* (represented by 28 individual genomes including two *M. insignis* genomes) and two *Liriodendron* species. Genes are denoted by colored boxes. The gaps between the genes and the boundaries are indicated by the base lengths (bp).

The size variation of angiosperm plastid genomes is primarily due to expansion and contraction of the IR and SSC boundary regions [53,55]. Detailed comparisons of the IR/SSC and IR/LSC junction sites between the *Magnolia* and *Liriodendron* species are presented in Figure 3. Generally, the IRA/LSC border is located between the *rpl2* and *trnH* genes in *Magnolia,* with *rpl2* in IRA and *trnH* in LSC, like that found in *Rosa roxburghii* Tratt. and *Rosa odorata* (Andr.) Sweet var. *gigantea* (Crép) Rehd. et Wils. [38]. The *trnH* gene of *M. zenii* extends only eight bp in length from LSC to IRA. The lengths of the LSC, IR, and SSC regions were similar in the plastomes of the 30 species studied. The JLB (IRB/LSC) border was located in the coding region of *rps19*. The *ycf1* gene spanned the JSA (SSC/IRA) region, and its length also reflected changes in the JSA region. *Magnolia dandyi* Gagnep. had the longest *ycf1* sequence with 5630 bp, while *Liriodendron chinense* (Hemsl.) Sarg. had the shortest sequence of 5489 bp. The *NdhF* gene deviated from the JSB (IRB/SSC) region in the species studied, ranging from 51 bp in *Magnolia acuminata* (L.) L. [56] to 93 bp in *M. officinalis* [30]. Indeed, *ycf1* was considered to have a close relationship to the position of the IR border in previous studies of Magnoliaceae species [30]. The tRNA gene *trnH-GUG* was located in the LSC, which ranged from 8–24 bp from the JLA (IRA/LSC) border. Apart from the ribosomal operon, IR gene content varies among angiosperms due to expansion and contraction of the IR/SC boundaries. Although many studies have reported changes in plastid genomes, including gene loss [57,58], inversion or deletions [59,60], and expansion or contraction of the IR region [61], in this study, IR boundary shifts tended to be relatively minor, involving just a small number of genes, and the position of the *trnH* gene located at the junction of IRA/LSC only presented irregular shifting. The highest divergence in non-coding regions was located in the intergenic regions of *rps16-trnQ*, *trnS-trnG*, and *ndhF-rpl32*. These regions were also identified as phylogenetically informative for studying the classification and evolutionary drift in other plant groups [62,63].

### 2.6. Phylogenetic Inference

**Figure 4:**
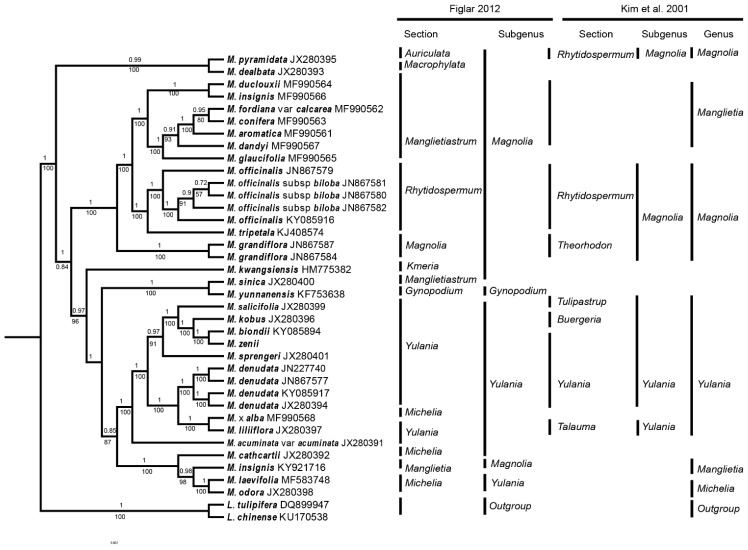
BI phylogenomic tree of 28 *Magnolia* (represented by 36 individuals) and 2 *Liriodendron* species. Numbers above the lines indicate the posterior probability of each clade, and numbers below the lines indicate the likelihood bootstrap values. The vertical lines on the right of the phylogenomic tree show the subgeneric taxonomic placement of these species according to Kim et al. [21] and Figlar [64].

To examine the phylogenetic position of *M. zenii* within the *Magnolia* genus, we selected complete plastid genome sequences from the plastomes of 28 *Magnolia* (represented by 36 individual sequences) and two *Liriodendron* species. In the ML and BI trees, most of the nodes had 100% bootstrap support and 1.0 Bayesian posterior probability (Figure 4 and Figure S2). BI analysis found that 25 of 36 nodes had posterior probability values of ≥99% (Figure 4), and 26 of these 36 nodes had 100% bootstrap support in ML analyses (Figure S2). Both the ML and BI trees had identical phylogenetic topologies, which strongly support the position of *M. zenii* as a sister to *M. biondii* in the subgenus *Yulania* (Figure 4). This position of *M. zenii* is in line with the previously-published phylogeny of Kim et al. [20,21], while Figlar [64] only provided a broad classification, placing *M. zenii* in section *Yulania*, and did not discuss relationships between species. Phylogenetic research using nuclear datasets [22] also found *M. zenii* to be nested within section *Yulania,* but placed as a sister to a clade comprising *M. denudata* Desr., *M. liliiflora*, *M. dawsoniana* Rehder & E.H. Wilson, and *M. sprengeri* Pamp. It is interesting to note, however, that the Flora of China [16] states *M. elliptigemmata* C.L. Guo & L.L. Huang to be the species most similar morphologically to *M. zenii*, although no molecular samples exist for *M. elliptigemmata* to explore its phylogenetic relationships.

Furthermore, early-diverging species, *M. pyramidata* W. Bartram and *M. dealbata* Zucc., belonged to sections *Auriculata* Figlar & Noot. and *Macrophylla* Figlar & Noot., respectively. These two species, both endemic to North America, formed monophyletic clades with 100% bootstrap support in the ML analyses (Figure S2) and 0.99 Bayesian posterior probability in BI analyses. In addition, *M. insignis* was placed as a sister to section *Michelia* with 96% posterior probability and bootstrap support in both the BI and ML trees, which conflicts with the results of Kim et al. [21]. As species previously treated as *Michelia*, i.e., *Magnolia alba* (DC.) Figlar and *M. odora* (Chun) Figlar & Noot., were found to be placed in different parts within the *Yulania* clade, this supports Figlar et al. [19], sinking *Michelia* into *Magnolia*, but does not corroborate the recognition of subgenus *Michelia* [22]. Although our taxon sampling was insufficient to perform a robust phylogenetic analysis of *Magnolia*, our data do provide information for future phylogenetic studies within the genus. Our discovery of gene regions with high polymorphism not used by previous phylogenetic studies in *Magnolia* [11,20,21,22,65] provides exciting avenues for future phylogenetic and evolutionary studies in this genus. It is now necessary that work be done to design the markers for these mutation hotspots to test their value in molecular studies.

## 3. Materials and Methods

### 3.1. DNA Extraction and Sequencing

Fresh *M. zenii* leaves were collected from one of the three *M. zenii* trees growing in the Nanjing botanical garden, Jiangsu province, China. Total genomic DNA was extracted using the modified cetyl trimethyl ammonium bromide (CTAB) method [66]. Following this, the sample genomic DNA was fragmented by mechanical disruption (ultrasound), and then, the fragmented DNA was subjected to fragment purification, end repair, 3′ end plus A, and ligation sequencing, and then, agarose gel electrophoresis was used to detect DNA integrity and quality. The fragment size was selected by electrophoresis, and the sequencing library was formed by PCR amplification. The constructed library was first subjected to library quality inspection, and the qualified library was sequenced by Illumina HiSeq platform technology. The quality and quantity of the genomic DNA were evaluated using the Nanodrop detection method. The experimental process was carried out in accordance with the standard protocol provided by the manufacturer’s instructions (Illumina Biotechnology company, San Diego, CA, USA), including sample quality testing, library construction, library quality testing, and library sequencing. Then, 41,103,536 raw reads were sequenced with paired-end (PE) 150 bp-length reads.

### 3.2. Chloroplast Genome Assembling and Annotation

Raw data were filtered, and high-quality clean data were obtained by removing the connector sequence and low-quality reads. The plastome was assembled via NOVOPlasty [67], with published *Magnolia* species as references. The annotation of rRNAs, tRNAs, and protein-coding genes of the plastome was performed using the CpGAVAS pipeline [68] and then manually corrected. OGDRAW [69] was used to visualize the structural features of the *M. zenii* plastome. The relative synonymous codon usage (RSCU) was examined using CodonW [70]. The annotated plastid genome sequence of *M. zenii* was deposited in GenBank (Accession number MH607378).

### 3.3. Whole Plastid Genome Comparison

To research genome-wide evolutionary dynamics in *Magnolia* and *Liriodendron* to reconstruct evolutionary events such as gene loss, duplication, rearrangement, and horizontal transfer, multiple genome alignments were done using Mauve software [71]. mVISTA [72,73] was used to find conserved sequences for functionally-significant regions. Thirty plastomes, including 28 *Magnolia* and 2 *Liriodendron* species were compared by the Shuffle-LAGAN alignment algorithm [74], using the plastome of *Magnolia laevifolia* (Y.W. Law & Y.F. Wu) Noot. as a reference. Irscope [75] was used to draw the genetic architecture of 10 *Magnolia* genomes in the junction of the sites connecting the IR regions to the LSC and SSC regions. DNA sequence variation within *Magnolia* was calculated by the DnaSP program [76] with the sliding window method in noncoding, synonymous, or nonsynonymous sites.

### 3.4. Repeat Structure Identification

The online software Reputer [77,78] was used to identify the repeat sequences (n ≥ 30 bp; sequence identity ≥90%). Four matches of repeats were classified into the following categories: (i) forward (direct) match, (ii) reverse match, (iii) complement match, and (iv) palindromic match. Simple sequence repeats (SSRs) were examined by Perlscript MISA [79] to detect mono-, di-, or tri-microsatellites.

### 3.5. Phylogenetic Analysis

Phylogenies were constructed by maximum likelihood (ML) and Bayesian inference (BI) analysis using 38 plastomes of 28 *Magnolia* (represented by 36 individuals) and 2 *Liriodendron* species (Figure 4, Figure S2, Table S1). The sequences were initially aligned using MAFFT [80] and then visualized and manually adjusted using BioEdit [81]. JmodelTest2 [82] was used to evaluate and select the best-fitting models of nucleotide sequences. A General Time Reversible + Proportion Invariation + Gamma nucleotide substitution model (GTR + I + G) were selected as the best substitution models for the ML and BI analyses. The Akaike information criterion [83] was used to select among models instead of the hierarchical likelihood ratio test, following Pol [84] and Posada and Buckley [85]. ML analyses were conducted in RaxML-HPC2 on TG ver. 7.2.8 on the Cipres web server [86,87], with 1000 rapid bootstrap analyses along with a search for the best-scoring tree in every run. BI analyses were conducted using Mrbayes v 3.2.6 [88], with two independent Markov chain Monte Carlo (MCMC) analyses run, each with three heated and one cold chain for 1,000,000 generations. Each chain started with a random tree; priors were set to default; and trees were sampled every 100 generations, with the first 25% discarded as burn-in. Calculations of the consensus tree, including clade posterior probability (PP), were performed based on the trees sampled after the chains converged.

## 4. Conclusions

Using Illumina high-throughput sequencing technology, we obtained the first complete plastome sequence of the critically endangered species, *M. zenii*. The plastid genome had a typical quadripartite structure with a conserved arrangement, the gene content and gene order being consistent with those found in plastomes of magnoliids and other typical angiosperms. Comparison of genome structure suggested that variation in the plastid genome structure of Magnoliaceae taxa is very limited, with almost no gene inversion or rearrangement found, which is consistent with previous studies [30]. This highly conserved plastome content implies that the plastid genome evolves very slowly in Magnoliaceae species, with *M. zenii* and its congeners a good study system for future investigation over the possible reasons for this slow genome evolution. While determining the distribution and location of repeat sequences, five regions of mutation hotspots at the level of genus and tribe were detected, which will be valuable for future molecular studies. Future research should now be conducted to design the markers for these mutation hotspots to test their value in phylogenetic studies. This is especially pertinent seeing as subgeneric relationships within *Magnolia* remain contentious, with the genus found to be paraphyletic and support along the backbone of the *Magnolia* phylogeny is poor [20,21,22]. The ML and BI phylogenetic trees strongly supported *M. zenii* as a member of the section *Yulania* and as a sister to *M. biondii*, but further studies should now be done using a more comprehensive taxon sampling that includes, e.g., morphologically-similar species such as *M. elliptigemmata* [15], to elucidate the classification and evolutionary history of this species.

The repeat motifs and mutation hotspots identified in the plastome can now be used to develop markers for population genetic studies and pave the way for more applied research focused on the monitoring and conservation of genetic diversity in wild populations of *M. zenii* that will be essential to the species’ long-term survival [89]. This unique tree has much horticultural and medicinal value, with there being great potential for conservation practices to bring the species back from the brink of extinction, including sourcing propagules for threatened species reintroductions and ecological restoration [90]. However, such strategies require an understanding of small population processes and inbreeding depression, local adaptation, and outbreeding depression [90,91], which can only be gained through careful monitoring of the species genetic resources, with our study providing important information to facilitate this.

## Figures and Tables

**Table 1:** List of genes annotated in the plastomes of *M. zenii* sequenced in this study.

Category		Name of Group		Name of Gene		
Self-replication	Ribosomal RNA	*rrn16*	*rrn23*	*rrn4.5*	*rrn5*	
	Transfer RNA	*trnA-UGC*	*trnC-GCA*	*trnD-GUC*	*trnE-UUC*	*trnF-GAA*
		*trnfM-CAU*	*trnG-GCC*	*trnG-UCC*	*trnH-GUG*	*trnI-CAU*
		*trnI-GAU*	*trnK-UUU*	*trnL-CAA*	*trnL-UAA*	*trnL-UAG*
		*trnM-CAU*	*trnN-GUU*	*trnP-UGG*	*trnQ-UUG*	*trnR-ACG*
		*trnR-UCU*	*trnS-GCU*	*trnS-GGA*	*trnS-UGA*	*trnT-GGU*
		*trnT-UGU*	*trnV-GAC*	*trnV-UAC*	*trnW-CCA*	*trnY-GUA*
	Small subunit of ribosome	*rps11*	*rps12*	*rps14*	*rps15*	*rps16*
		*rps18*	*rps19*	*rps2*	*rps3*	*rps4*
		*rps7*	*rps8*			
	Large subunit of ribosome	*rpl12*	*rpl14*	*rpl16*	*rpl2*	*rpl20*
		*rpl23*	*rpl32*	*rpl33*	*rpl36*	
	RNA polymerase subunits	*rpoA*	*rpoB*	*rpoC1*	*rpoC2*	
Photosynthesis	Subunits of photosystem I	*psaA*	*psaB*	*psaC*	*psaI*	*psaJ*
	Subunits of photosystem II	*psbA*	*psbB*	*psbC*	*psbD*	*psbE*
		*psbF*	*psbH*	*psbI*	*psbJ*	*psbK*
		*psbL*	*psbM*	*psbN*	*psbT*	*psbz*
	Subunits of cytochrome	*petA*	*petB*	*petD*	*petG*	*petL*
		*petN*				
	Subunits of ATP synthase	*atpA*	*atpB*	*atpE*	*atpF*	*atpH*
		*atpI*				
	Large subunit of RuBisCO	*rbcL*				
	Subunits of NADH	*ndhA*	*ndhB*	*ndhC*	*ndhD*	*ndhE*
		*ndhF*	*ndhG*	*ndhH*	*ndhI*	*ndhJ*
		*ndhK*				
Other gene	Translational initiation factor	*infA*				
	Maturase	*matK*				
	Envelope membrane protein	*cemA*				
	Subunit of acetyl-CoA	*accD*				
	C-type cytochrome synthesis gene	*ccsA*				
	Protease	*clpP*				
Unknown function	Conserved open reading frames	*ycf1*	*ycf15*	*ycf2*	*ycf3*	*ycf4*

## Supplementary Materials

The following are available online: Table S1: Summary of the plastid genome features of the 28 *Magnolia* and 2 *Liriodendron* species studied, Table S2: Base composition in the *M. zenii* plastid genome, Table S3: The length of exons and introns in genes with introns in the *M. zenii* plastid genome, Table S4: Codon-anticodon recognition patterns and codon usage of the *M. zenii* plastid genome, Table S5: Simple sequence repeats (SSRs) in the *M. zenii* plastid genome, Table S6. Long repeat sequence in the *M. zenii* plastid genome, Figure S1: Complete plastid genome comparison of 28 *Magnolia* and two *Liriodendron* species using the plastome of *M. laevifolia* as a reference. The grey arrows and thick black lines above the alignment indicate the genes’ orientation. The y-axis represents the percent identity between 50 and 100%. Red and blue areas indicate intergenic and genic regions, respectively, Figure S2: ML phylogenomic tree of 28 *Magnolia* (represented by 36 individuals) and 2 *Liriodendron* species. Numbers above the lines indicate the bootstrap support of each clade when >50%.
