# Supplementary material for: The Complete Plastid Genome of Magnolia zenii and Genetic Comparison to Magnoliaceae species
Source: Molecules. 2019 Jan 11;24(2):261. doi: 10.3390/molecules24020261 (PMC6359370; doi:10.3390/molecules24020261)
Supplement: Supplementary file 1 [file molecules-24-00261-s001.pdf]

molecules

MDPI

Table S1. Summary of the plastid genome features of the 28 *Magnolia* and 2 *Liriodendron* species studied.

Species	Accession	Size (bp)	LSC length (bp)	SSC length (bp)	IR length (bp)	Protein-coding genes	tRNA genes	rRNA genes	Number of genes	Overall GC content (%)	GC content of LSC (%)	GC content of SSC (%)	GC content of IR (%)
Magnolia acuminata (L.) L. var. *acuminata*	JX280391.1	159812	87838	18770	26602	86	37	8	131	39.3	38	34	43
Magnolia × alba (DC.) Figlar	MF990568.1	159789	87951	18798	26570	83	68	8	156	39	38	34	43
Magnolia aromatica (Dandy) V.S.Kumar	MF990561.1	160062	88087	18831	26572	86	37	8	131	39.3	38	34.2	43.2
Magnolia biondii Pamp.	KY085894.1	160002	88075	18795	26566	84	37	8	129	39.2	38	34	43
Magnolia cathcartii (Hook.f. & Thomson) Noot.	JX280392.1	159950	88142	18790	26509	86	37	8	131	39.2	38	34	43
Magnolia conifera (Dandy) V.S.Kumar	MF990563.1	159973	88088	18741	26572	86	37	8	132	39	38	34.3	43.2
Magnolia dandyi Gagnep.	MF990567.1	160077	88095	18838	26572	86	37	8	131	39.3	38	34	43
Magnolia dealbata Zucc.	JX280393.1	160002	88088	18740	26587	86	36	8	130	39.2	38	34	43
Magnolia denudata Desr.	JN227740.1	160053	88101	18772	26590	84	37	8	129	39.3	38	34	43
Magnolia duclouxii Hu	MF990564.1	160055	88118	18789	26574	86	37	8	131	39.3	38	34.3	43.2
Magnolia fordiana Hu var. *calcarea*	MF990562.1	160027	88088	18795	26572	86	37	8	131	39.3	38	34.2	43.2

(X.H.Song) V.S.Kumar												
Magnolia glaucifolia (Y.W.Law & Y.F.Wu) Noot.	MF990565.1	160059	88094	18803	26581	86	37	8	131	39.3	38	34.3	43.2
Magnolia grandiflora L.	NC_020318	159623	87757	18740	26563	84	37	8	129	39	38	38	42
Magnolia insignis Wall.	KY921716.1	160117	88307	18660	26575	86	37	4	131	39	39	34	43
Magnolia kobus DC.	JX280396.1	159443	87484	18783	26588	79	30	4	113	39.28	38	34	43
Magnolia kwangsiensis Figlar & Noot.	HM775382.1	159667	88030	18669	26484	84	37	8	129	39	38	34	43
Magnolia laevifolia	MF583748.1	160120	88145	18799	26588	86	37	8	131	39	37	34	43
Magnolia liliiflora Desr.	JX280397.1	158177	88133	18742	26588	86	35	8	129	39.1	38	34	43
Magnolia odora (Chun) Figlar & Noot.	JX280398.1	160070	88098	18800	26586	86	37	8	131	39.3	38	34	43
Magnolia officinalis Rehder & E.H.Wilson	JN867579.1	160183	88210	18843	26565	81	37	8	126	39	38	34	43
Magnolia pyramidata W. Bartram	JX280395.1	160025	88043	18788	26597	86	37	8	131	39.2	38	34	43
Magnolia salicifolia Maxim.	JX280399.1	160093	88143	18768	26591	86	37	8	131	39.3	38	34	43
Magnolia sinica (Law Yuh-wu) Noot.	JX280400.1	160044	88156	18746	26571	86	37	8	131	39.3	38	34	43
Magnolia sprengeri Pamp.	JX280401.1	160033	88133	18722	26589	86	37	8	131	39.2	38	34	43

Magnolia tripetala	KJ408574.1	160037	88148	18745	26572	87	37	8	132	39.3	38	34	43
Magnolia yunnanensis (Hu) Noot.	KF753638.1	160085	88170	18745	26585	91	37	8	131	39.3	37.9	34.3	43.2
Magnolia zenii		160048	88098	18757	26596	84	37	8	130	39	38	34	43
Liriodendron chinense (Hemsl.) Sarg.	KU170538.1	159429	87766	18997	26333	79	30	4	113	39	38	34	43
Liriodendron tulipifera L.	DQ899947.1	159886	88150	18964	26386	84	37	8	129	39	38	34	43

Table S2. Base composition in the *M. zenii* plastid genome.

Region	A (%)	T (U) (%)	C (%)	G (%)	A + T (%)	C + G (%)
LSC	30.33781	31.72263	19.42155	18.51801	62.06043	37.93957
SSC	32.90863	32.91396	18.05096	16.12645	65.82258	34.17742
IRA	28.57949	28.25237	20.85276	22.31539	56.83185	43.16815
IRB	28.25237	28.57949	22.31539	20.85276	56.83185	43.16815
Total	30.01957	30.36711	20.16017	19.45315	60.38668	39.61332

Table S3. The length of exons and introns in genes with introns in the *M. zenii* plastid genome.

Gene	Location	Exon I (bp)	Intron I (bp)	Exon II (bp)	Intron II (bp)	Exon III (bp)
trnK	LSC	37	2,490	35		
rps16	LSC	44	825	217		
trnG	LSC	24	767	48		
atpF	LSC	144	708	411		
rpoC1	LSC	434	722	1,624		
ycf3	LSC	126	739	228	729	153
trnL	LSC	35	495	50		
trnV	LSC	39	584	37		
rps12	LSC	114				
clpP	LSC	69	781	291	628	246
rpl2	IRA	384	661	432		30
ndhB	IRA	777	700	756		
rps12	IRA		537	228		30
trnI	IRA	42	936	35		
trnA	IRA	38	799	35		
ndhA	SSC	551	1,075	541		
trnA	IRB	35	799	38		
trnI	IRB	36	935	43		
rps12	IRB		533	228		30
ndhB	IRB	777	700	756		
rpl2	IRB	384	661	432		

Table S4. Codon-anticodon recognition patterns and condon usage of the *M. zenii* plastid genome.

Amino Acid	Codon	No.	RSCU	tRNA	Amino Acid	Codon	No.	RSCU	tRNA
Phe	UUU	805	1.14		Ser	UCU	575	1.64	
	UUC	603	0.86	trnF-GAA		UCC	359	1.02	trnS-GGA
Leu	UUA	726	1.62			UCA	458	1.3	trnS-TGA
	UUG	580	1.3	trnL-CAA		UCG	209	0.59	
	CUU	554	1.24		Pro	CCU	422	1.5	
	CUC	203	0.45			CCC	244	0.87	
	CUA	407	0.91	trnL-TAG		CCA	331	1.17	trnP-TGG
	CUG	211	0.47			CCG	130	0.46	
Ile	AUU	1042	1.41		Thr	ACU	519	1.52	
	AUC	512	0.69			ACC	272	0.8	trnT-GGT
	AUA	662	0.9			ACA	416	1.22	trnT-TGT
Met	AUG	623	1	trnM-CAT		ACG	155	0.46	
Val	GUU	508	1.39		Ala	GCU	635	1.84	
	GUC	193	0.53	trnV-GAC		GCC	219	0.64	
	GUA	519	1.42			GCA	389	1.13	
	GUG	237	0.65			GCG	134	0.39	
Tyr	UAU	741	1.58		Cys	UGU	231	1.5	
	UAC	199	0.42	trnY-GTA		UGC	76	0.5	trnC-GCA
TER	UAA	33	1.16		TER	UGA	26	0.92	
	UAG	26	0.92		Trp	UGG	468	1	trnW-CCA
His	CAU	497	1.5		Arg	CGU	366	1.34	trnR-ACG
	CAC	166	0.5	trnH-GTG		CGC	87	0.32	
Gln	CAA	673	1.45	trnQ-TTG		CGA	373	1.37	
	CAG	255	0.55			CGG	127	0.47	
Asn	AAU	951	1.53		Ser	AGU	401	1.14	
	AAC	290	0.47	trnN-GTT		AGC	107	0.3	trnS-GCT

Lys	AAA	934	1.43		Arg	AGA	506	1.86	trnR-TCT
	AAG	371	0.57			AGG	175	0.64	
Asp	GAU	903	1.6		Gly	GGU	597	1.31	
	GAC	225	0.4	trnD-GTC		GGC	171	0.38	trnG-GCC
Glu	GAA	995	1.43	trnE-TTC		GGA	744	1.64	
	GAG	395	0.57			GGG	305	0.67	

Table S5. Simple sequence repeats (SSRs) in the *M. zenii* plastid genome.

Gene ID	SSR nr	SSR type	SSR Size	Start	End	Gene ID	SSR nr	SSR type	SSR Size	Start	End
1	p1	(A)11	11	211	221	20	p1	(A)12	12	38882	38893
2	c	(A)10 (T)15	53	1727	1779	21	p1	(A)14	14	46923	46936
3	p1	(A)10	10	3947	3956	22	p1	(A)10	10	49856	49865
4	p1	(T)11	11	4590	4600	23	p1	(T)11	11	53533	53543
5	p1	(A)10	10	4758	4767	24	p1	(T)10	10	55856	55865
6	c	(T)12(A)10	31	5385	5415	25	p1	(T)10	10	64382	64391
7	p1	(C)13	13	7185	7197	26	p1	(A)11	11	64740	64750
8	p1	(T)11	11	7765	7775	27	p1	(C)13	13	71220	71232
9	p1	(T)11	11	10253	10263	28	p2	(TA)6	12	71834	71845
10	p1	(A)12	12	12752	12763	29	p1	(T)11	11	72362	72372
11	p1	(A)11	11	13840	13850	30	p1	(T)12	12	75472	75483
12	c	(T)10(A)12	23	15805	15827	31	p1	(T)11	11	84455	84465
13	p1	(T)11	11	17536	17546	32	p1	(T)11	11	84953	84963
14	p1	(T)12	12	19734	19745	33	c	(C)11 (A)10	21	118569	118589
15	p1	(A)11	11	29204	29214	34	p1	(T)12	12	124188	124199
16	p1	(A)10	10	30572	30581	35	p1	(A)10	10	126119	126128
17	p1	(T)11	11	31359	31369	36	p2	(TC)6	12	128806	128817
18	p1	(T)10	10	33496	33505	37	p1	(A)10	10	132539	132548
19	p2	(TA)6	12	34992	35003						

Table S6. Long repeat sequences in the *M. zenii* plastid genome.

Match direction	Size of repeat I	Repeat start I	Location	Repeat start II	Size of repeat II	Location	Distance of repeat	E-value
F	57	132739	ycf1	132757	57	ycf1	0	3.47E-25
F	39	132739	ycf1	132775	39	ycf1	0	2.38E-14
F	42	92849	ycf2	92870	42	ycf2	−1	4.69E-14
P	42	92849	ycf2	155234	42	ycf2	−1	4.69E-14
P	42	92870	ycf2	155255	42	ycf2	−1	4.69E-14
F	42	155234	IGS	155255	42	ycf2	−1	4.69E-14
P	45	15793	IGS	15793	45	IGS	−3	2.23E-12
P	42	130305	ycf1	130305	42	ycf1	−2	2.89E-12
F	33	95283	ycf2	95307	33	ycf2	0	9.76E-11
P	33	95283	ycf2	152806	33	ycf2	0	9.76E-11
P	33	95307	ycf2	152830	33	ycf2	0	9.76E-11
F	33	152806	ycf2	152830	33	ycf2	0	9.76E-11
F	39	46248	ycf3	102716	39	ycf3	−2	1.59E-10
P	39	46248	ycf3	145391	39	ycf3	−2	1.59E-10
P	32	67797	IGS	67841	32	IGS	0	3.91E-10
F	41	41297	psaB	43521	41	psaB	−3	4.29E-10
F	31	155245	ycf2	155266	31	ycf2	0	1.56E-09
P	37	128925	IGS	128925	37	IGS	−3	8.00E-08
P	30	9263	trnS-GGA	47565	30	trnS-GGA	−1	5.62E-07
F	31	9262	IGS	38107	31	IGS	−2	6.54E-06
P	33	31566	IGS	31566	33	IGS	−3	1.44E-05

P	30	38108	trnS-UGA	47565	30	trnS-GGA	−3	6.85E-04
F	30	46260	ycf3	102728	30	IGS	−3	6.85E-04
P	30	46260	ycf3	145388	30	IGS	−3	6.85E-04
F	30	113246	IGS	113287	30	IGS	−3	6.85E-04
P	30	113246	IGS	134829	30	IGS	−3	6.85E-04
P	30	113287	IGS	134870	30	IGS	−3	6.85E-04
F	30	134831	IGS	134872	30	IGS	−3	6.85E-04

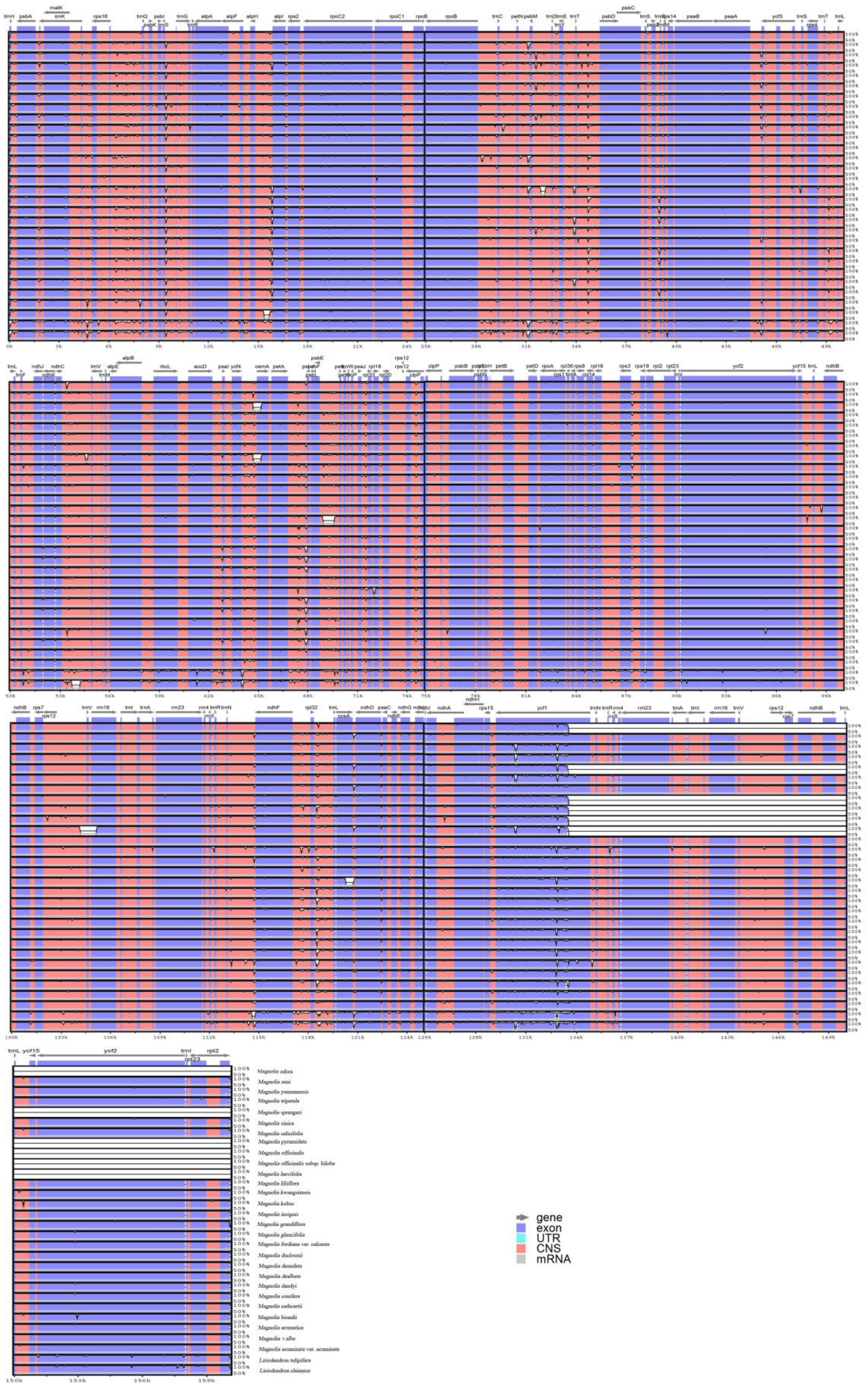

Figure S1. Complete plastid genome comparison of 28 *Magnolia* and two *Liriodendron* species using the plastome of *M. laevifolia* as a reference. The grey arrows and thick black lines above

the alignment indicate the genes' orientation. The y-axis represents the percent identity between 50-100%. Red and blue areas indicate intergenic and genic regions, respectively.

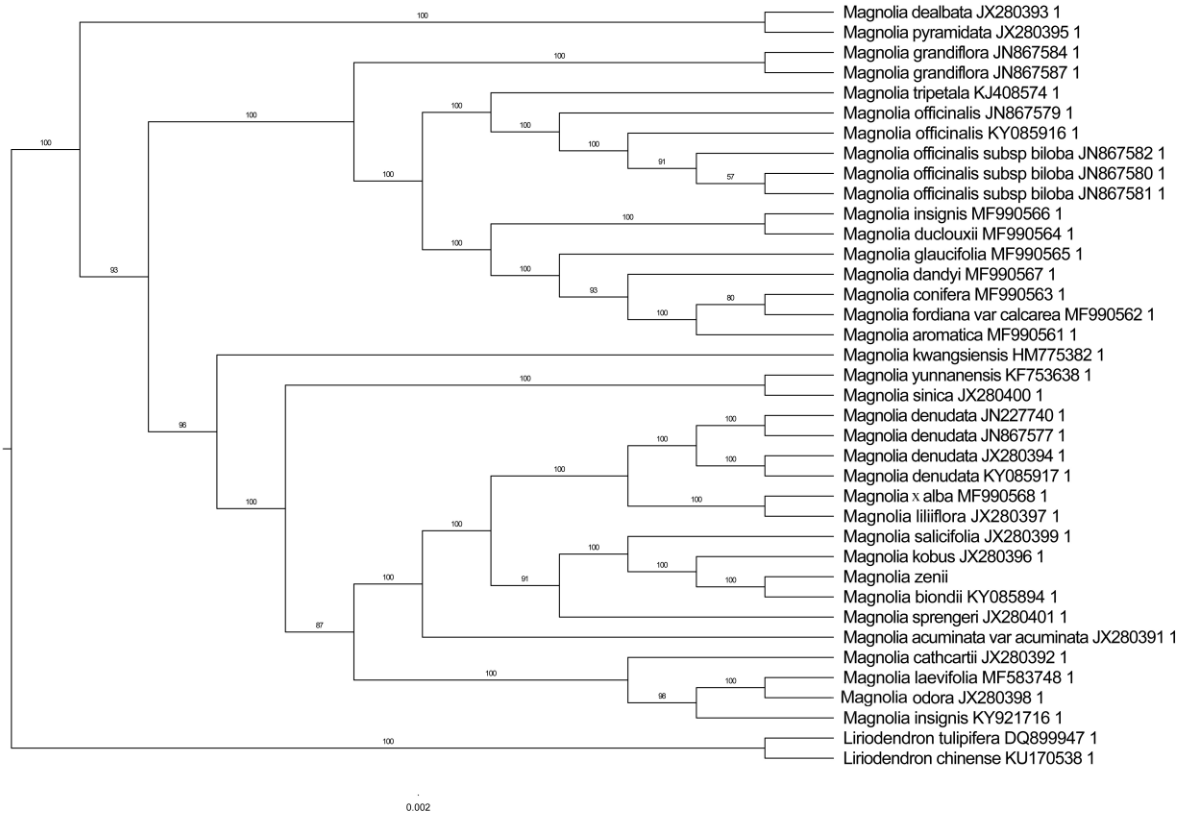

Figure S2. ML phylogenomic tree of 28 *Magnolia* (represented by 36 individuals) and 2 *Liriodendron* species. Numbers above the lines indicate the bootstrap support of each clade when >50%.
